# Effect of delayed cord clamping on maternal and neonatal outcome in twin pregnancies: a retrospective cohort study

**DOI:** 10.1038/s41598-023-44575-9

**Published:** 2023-10-13

**Authors:** Suin Yoon, Yookyung Jin, Yejin Kim, Ji-Hee Sung, Suk-Joo Choi, Soo-young Oh, Cheong-Rae Roh

**Affiliations:** Department of Obstetrics and Gynecology, Samsung Medical Center, Sungkyunkwan University School of Medicine, 81 Irwon-ro, Gangnam-gu, Seoul, 06351 Korea

**Keywords:** Health care, Medical research

## Abstract

The objective of this study was to compare the maternal and neonatal outcomes following delayed cord clamping (DCC) versus immediate cord clamping (ICC) in twin pregnancies. This was a retrospective cohort study of 705 twin pregnancies who delivered at ≥ 24 weeks of gestation. Maternal and neonatal hemoglobin levels, blood transfusion, and neonatal outcomes were compared between DCC (n = 225) and ICC (n = 480) groups. Mean maternal predelivery and postpartum hemoglobin levels and the rate of postpartum hemoglobin drop ≥ 20% or maternal blood transfusion were comparable between the two groups. The DCC group had a significantly higher mean neonatal hemoglobin level (DCC vs. ICC: 17.4 ± 3.5 vs. 16.6 ± 2.7 g/dl, P = 0.010) but significantly lower rates of neonatal blood transfusion (DCC vs. ICC: 3.3% vs. 8.8%, P < 0.001) and respiratory distress syndrome (DCC vs. ICC: 6.7% vs. 15.2%, P < 0.001) than the ICC group. In conclusion, DCC compared with ICC in twin pregnancy was not associated with an increase of maternal postpartum bleeding complications, but it was associated with higher neonatal hemoglobin level and lower risks of neonatal blood transfusion and respiratory distress syndrome.

## Introduction

Delayed cord clamping (DCC) is defined as umbilical cord clamping delayed for at least 30‒60 s after birth. DCC allows placental transfusion of warm, oxygenated blood to flow into newborn. It is known to be more beneficial for term and preterm infants than traditional immediate cord clamping (ICC). In term infants, DCC can increase hematocrit/hemoglobin levels at birth and during neonatal period and reduce the frequency of iron-deficiency anemia in the first several months of life^1–3^. In preterm infants, DCC can reduce the need for blood transfusion, the frequency of respiratory distress syndrome, intraventricular hemorrhage, necrotizing enterocolitis^1,3–5^. However, DCC might have potential risks of neonatal polycythemia, hyperbilirubinemia, jaundice^6^, and maternal blood loss from episiotomy site or uterine incision site, especially in multiple pregnancies^6,7^.

Given the trend of increasing maternal age and increasing use of assisted reproductive technology (ART), the frequency of multiple pregnancies has gradually increased during the last few decades^8,9^. Several randomized controlled trials have been proven that DCC is beneficial for both term and preterm singleton pregnancies^2,10,11^. However, benefits and risks of DCC in multiple pregnancies are not well established yet. Although there have been a few studies on the effect of DCC in multiple pregnancies^6,7,12–16^, most of these studies included only a small number of subjects with different study populations. Specifically, the only one randomized controlled trial included only 47 twin and triplet pregnancies^7^. Other retrospective cohort studies included only preterm twins^6,12,14,15^ or dichorionic twins^6,14^. Some studies were focused on neonatal outcome only^12,14–16^ or maternal outcome only^13^. Moreover, results of these studies are controversial and conflicting. Therefore, there are no sufficient evidences to recommend or against DCC in multiple pregnancies^17^.

Thus, the objective of the present study was to compare the maternal and neonatal outcomes following DCC versus ICC in all twin pregnancies including both preterm and term gestation.

## Methods

This was a retrospective cohort study including all twin pregnant women who delivered at ≥ 24 weeks of gestation between January 2014 and December 2021 in Samsung Medical Center, Seoul, South Korea. Inclusion criteria were twin pregnant women aged 18 years or older who had deliveries of both live twins at ≥ 24 weeks of gestation. Patients with fetal death, 1-min Apgar score < 4, or lethal congenital anomaly in one or more twins, combined vaginal and cesarean delivery, and DCC in only one twin were excluded. Subjects were categorized into two groups: umbilical cord clamping of both twin babies at 1 min after birth (DCC group) and umbilical cord clamping of both twin babies immediately after birth (ICC group). DCC or ICC was performed at physicians’ discretion. DCC in twin pregnancies was performed as follows. During vaginal delivery at preterm gestation, each twin baby was placed at the mother’s perineum level and umbilical cord clamping was done after 1 min of each baby’s delivery. During vaginal delivery at term gestation, each twin baby was delivered and placed on the mother’s abdomen because position of the baby before cord clamping did not affect volume of placental transfusion in a previous randomized study^18^. During the interval between the first and second twin delivery, active bleeding from the episiotomy site was compressed or ligated. During cesarean section at both preterm and term gestation, the first-born twin was placed on mother’s thigh without clamping the umbilical cord and the second was delivered immediately. Umbilical cord clamping was done at 1 min after each twin baby delivery. During the interval between the first twin delivery and second twin DCC, active bleeding from the uterine incision site was compressed or ligated. When the baby needed an immediate resuscitation after birth, umbilical cord clamping was done immediately. These cases were excluded from the analysis. This study was approved by the Institutional Review Board for Clinical Research at Samsung Medical Center (No. 2022-12-101) and exemption for informed consent was granted because this was a retrospective chart review study. All methods were carried out in accordance with relevant guidelines and regulations.

Maternal characteristics and obstetric and neonatal outcomes were obtained by reviewing their medical records. Maternal characteristics included age, parity, pre-pregnancy body weight, height, and body mass index (BMI), weight at delivery, history of preterm delivery (PTD), ART conception, and chorionicity. Chorionicity was evaluated by prenatal ultrasound and confirmed after delivery by obstetricians and pathology reports, where available. Pregnancy outcomes included antenatal corticosteroids treatment, preterm labor, incompetent internal os of cervix (IIOC), preterm premature rupture of membrane (PPROM), preeclampsia, placenta previa, placenta abruption, gestational diabetes, gestational age at delivery, PTD < 34 weeks and < 37 weeks of gestation, cesarean section, indications for cesarean section, predelivery and postpartum hemoglobin levels, and blood transfusion.

Neonatal outcomes included birth weight, sex, 1-min and 5-min Apgar scores, hemoglobin level at birth, blood transfusion, neonatal intensive care unit (NICU) admission, mechanical ventilation therapy, respiratory distress syndrome (RDS), bronchopulmonary dysplasia (BPD), transient tachypnea of the newborn (TTN), intraventricular hemorrhage (IVH) (≥ grade 3), periventricular leukomalacia (PVL), necrotizing enterocolitis (NEC) (≥ stage 2), retinopathy of prematurity (ROP) (≥ stage 3), hypoglycemia, hyperbilirubinemia, neonatal sepsis, and neonatal mortality. Large-for-gestational age or small-for-gestational age were defined as neonatal birth weight > 90th or < 10th percentiles for gestational age, respectively, based on birth weight standards adjusted for gestational age and plurality from a Korean national database^19^.

Primary outcomes of this study were maternal postpartum hemoglobin levels, neonatal hemoglobin level at birth, and maternal and neonatal blood transfusion. Secondary outcomes were neonatal complications including NICU admission, mechanical ventilator therapy, RDS, BPD, TTN, IVH (≥ grade 3), PVL, NEC (≥ stage 2), ROP (≥ stage 3), hypoglycemia, hyperbilirubinemia, neonatal sepsis, and neonatal death. Two-sample Student’s *t*-test was performed to compare continuous variable and Chi-square test or Fisher's exact test was performed to compare categorical variables, as appropriate. To analyze the outcomes between the two groups that were not comparable in the baseline characteristics and important exposure variables, we performed subgroup analyses and multivariable analyses. For the primary outcomes (maternal and neonatal hemoglobin levels, blood transfusion, and maternal postpartum hemoglobin drop ≥ 20%), subgroup analyses were done by stratifying the patients according to gestational age (preterm vs. term), mode of delivery (vaginal delivery vs. cesarean section), and chorionicity (monochorionic vs. dichorionic). In addition, we performed subgroup analyses to compare neonatal hemoglobin levels and blood transfusion according to the twin birth order (DCC 1st twin vs. ICC 1st twin, and DCC 2nd twin vs. ICC 2nd twin). Multiple logistic regression analysis was performed to evaluate effects of potential confounding variables including parity, PPROM, antenatal corticosteroids treatment, chorionicity, gestational age at delivery, cesarean section, twin birth order, birth weight, sex, and SGA on neonatal complications that were significantly different in the univariable analysis. A two-tailed P-value less than 0.05 was considered statistically significant. All statistical analyses were carried out using the Statistical Package for Social Sciences version 25 (SPSS Statistics; IBM, Armonk, NY, USA).

### Ethical approval

This study was approved by the Institutional Review Board for Clinical Research at Samsung Medical Center at December 22, 2022 (IRB No. 2022-12-101).

## Results

**Figure 1:**
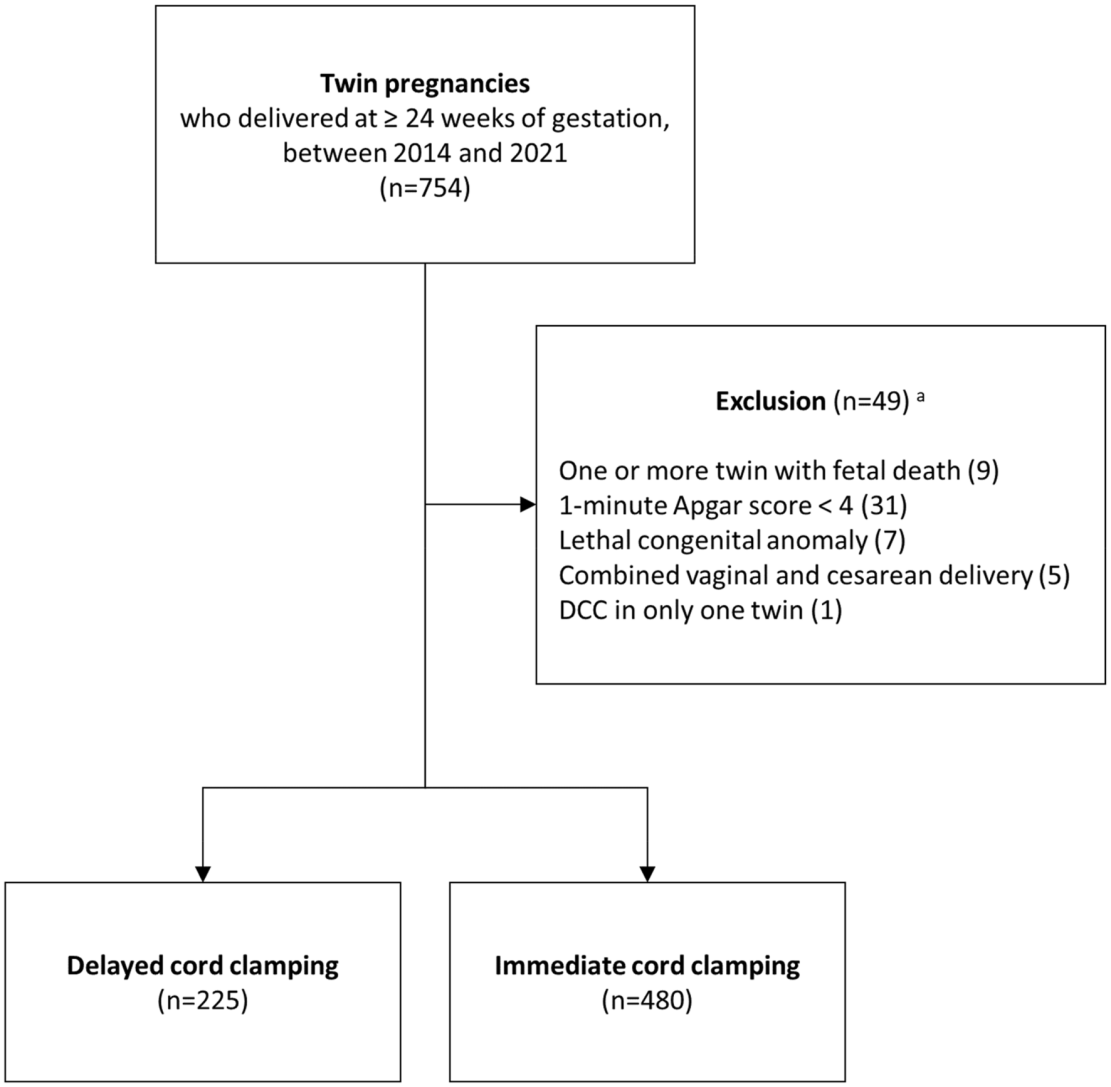
Flow chart showing the selection of patients in this study. ^a^Among 49 cases, 2 cases met the 2 exclusion criteria and 1 case met the 3 exclusion criteria at the same time.

During the study period, 754 women with twin pregnancies delivered at ≥ 24 weeks of gestation in our institute. After excluding 49 women based on our exclusion criteria, a total of 705 twin pregnant women were included in the final analysis (Fig. 1). DCC was performed in 225 (31.9%) of 705 women.

Maternal characteristics including age, BMI, ART pregnancy, and chorionicity were comparable between the two groups (Table 1). However, the proportion of multiparous women was significantly higher in the ICC group than in the DCC group. Rates of pregnancy complications including preterm labor, IIOC, preeclampsia, placenta previa, placenta abruption, and gestational diabetes were similar in the two groups (Table 2). However, the rates of antenatal corticosteroids treatment and PPROM were significantly higher in the ICC group than in the DCC group. Gestational age at delivery was significantly higher in the DCC group than in the ICC group. Rates of PTD and cesarean delivery were significantly higher in the ICC group than in the DCC group. Mean maternal predelivery hemoglobin levels were similar between the two groups (Table 4). Mean maternal postpartum hemoglobin levels on the first day (DCC vs. ICC: 10.3 ± 1.6 vs. 10.5 ± 1.6 g/dl, P = 0.111) and the third day (DCC vs. ICC: 9.9 ± 1.5 vs. 10.1 ± 1.5 g/dl, P = 0.346) were also comparable between the two groups. Rates of postpartum hemoglobin drop ≥ 20% and maternal blood transfusion were not significantly different between the two groups. In subgroup analyses, mean maternal predelivery and postpartum hemoglobin levels (day 1 and day 3) were also similar between DCC and ICC groups (Table 5). However, the rate of postpartum hemoglobin drop ≥ 20% was significantly higher in the DCC group than in the ICC group for those with a preterm delivery.

**Table 1:** Maternal characteristics.

Characteristics	Delayed cord clamping (n = 225)	Immediate cord clamping (n = 480)	*P-*value
Maternal age (year)	34.4 ± 3.9	34.1 ± 3.7	0.392
Maternal age ≥ 35 years	111 (49.3)	230 (47.9)	0.726
Height (cm)	162.3 ± 5.0	162.5 ± 5.5	0.642
Pre-pregnancy weight (kg)	56.4 ± 8.1	57.3 ± 10.4	0.288
Pre-pregnancy BMI (kg/m^2^)	21.4 ± 3.0	21.6 ± 3.7	0.395
Weight at delivery (kg)	71.8 ± 9.1	72.3 ± 12.5	0.581
BMI at delivery (kg/m^2^)	27.3 ± 3.3	27.4 ± 4.5	0.705
Multiparity	36 (16.0)	115 (24.0)	0.016
History of preterm delivery	6 (2.7)	19 (4.0)	0.387
ART pregnancy	137 (60.9)	284 (59.2)	0.648
Chorionicity			
Monoamnionic	0	1 (0.2)	0.478
Monochorionic diamnionic	31 (13.8)	75 (15.6)	
Dichorionic diamnionic	194 (86.2)	404 (84.2)	

Data are presented as number (percentage) or mean ± standard deviation.

*BMI* body mass index, *ART* assisted reproductive technology.

**Table 2:** Pregnancy outcomes.

Outcome variables	Delayed cord clamping (n = 225)	Immediate cord clamping (n = 480)	*P-*value
Antenatal corticosteroids treatment	56 (24.9)	197 (41.0)	< 0.001
Preterm labor	56 (24.9)	146 (30.4)	0.130
PPROM	25 (11.1)	106 (22.1)	< 0.001
IIOC	8 (3.6)	24 (5.0)	0.390
Preeclampsia	31 (13.8)	56 (11.7)	0.427
Placenta previa	8 (3.6)	11 (2.3)	0.334
Placenta abruptio	5 (2.2)	20 (4.2)	0.193
Gestational diabetes^a^	27/220 (12.3)	64/454 (14.1)	0.516
Gestational age at delivery (week)	36.0 ± 2.5	35.0 ± 3.5	< 0.001
Preterm delivery < 37 weeks	92 (40.9)	278 (57.9)	< 0.001
Preterm delivery < 34 weeks	36 (16.0)	125 (26.0)	0.003
Vaginal delivery	37 (16.4)	36 (7.5)	< 0.001
Cesarean section	188 (83.6)	444 (92.3)	
Indication for cesarean section			
Elective	40 (21.3)	100 (22.5)	0.237
Malpresentation	103 (54.8)	220 (49.5)	
Placenta previa	8 (4.3)	7 (1.6)	
Failure to progress	6 (3.2)	18 (4.1)	
Fetal indication	5 (2.7)	23 (5.2)	
Maternal indication	14 (7.4)	37 (8.3)	
Previous cesarean section or uterine surgery	12 (6.4)	39 (8.8)	

Data are presented as number (percentage) or mean ± standard deviation.

*PPROM* preterm premature rupture of membranes, *IIOC* incompetent internal os of cervix.

^a^Analyzed only cases with data available on gestational diabetes.

Birth weight was significantly higher in the DCC group. However, proportions of SGA, appropriate for gestational age (AGA), and LGA were not significantly different between the two groups (Table 3). There was no difference in the proportion of those with a 5-min Apgar score below 7 between the two groups. Neonatal hemoglobin levels were available for 151 and 433 cases in DCC and ICC groups, respectively. Mean neonatal hemoglobin level was significantly higher in the DCC group than in the ICC group (DCC vs. ICC: 17.4 ± 3.5 vs. 16.6 ± 2.7 mg/dl, P = 0.010) (Table 4). However, in subgroup analyses, mean neonatal hemoglobin level was significantly higher in the DCC group than in the ICC group only in the cesarean section group (Table 5). Neonatal blood transfusion data were available for all neonates. Neonatal blood transfusion rate was significantly lower in the DCC group than in the ICC group (3.3% vs. 8.8%, P < 0.001) (Table 4). In subgroup analyses, neonatal blood transfusion rate was significantly lower in the DCC group than in the ICC group in the preterm delivery group, cesarean section group, and dichorionic twin group (Table 5). In the subgroup analyses to compare the neonatal hemoglobin level and blood transfusion of according to the twin birth order (DCC 1st twin vs. ICC 1st twin, and DCC 2nd twin vs. ICC 2nd twin), overall results were similar to those of all twins (Table 6).

**Table 3:** Neonatal characteristics.

Characteristics	Delayed cord clamping (n = 450)	Immediate cord clamping (n = 960)	*P-*value
Gestational age at delivery (week)	36.0 ± 2.5	35.0 ± 3.5	< 0.001
Sex (male)	225 (50.0)	491 (51.1)	0.688
Birth weight (kg)	2.4 ± 0.5	2.2 ± 0.6	< 0.001
SGA	31 (6.9)	72 (7.5)	0.178
AGA	357 (79.3)	788 (82.1)	
LGA	62 (13.8)	100 (10.4)	
5-min Apgar score < 7	4 (0.9)	11 (1.1)	0.786

Data are presented as number (percentage) or mean ± standard deviation.

*SGA* small for gestational age, *AGA* appropriate for gestational age, *LGA* large for gestational age.

**Table 4:** Primary and secondary outcomes.

Outcome variables	Delayed cord clamping	Immediate cord clamping	*P-*value
Maternal outcomes	(n = 225)	(n = 480)	
Predelivery Hb level (g/dl)	11.8 ± 1.4	11.7 ± 1.5	0.457
Postpartum day 1 Hb level (g/dl)	10.3 ± 1.6	10.5 ± 1.6	0.111
Postpartum day 3 Hb level (g/dl)	9.9 ± 1.5	10.1 ± 1.5	0.346
Postpartum Hb drop ≥ 20%	92 (40.9)	161 (33.5)	0.058
Blood transfusion	20 (8.9)	35 (7.3)	0.461
Neonatal outcomes	(n = 450)	(n = 960)	
Neonatal Hb level at birth (g/dl)^a^	17.4 ± 3.5	16.6 ± 2.7	0.010
Neonatal blood transfusion	15 (3.3)	84 (8.8)	< 0.001
NICU admission	142 (31.6)	406 (42.3)	< 0.001
Mechanical ventilator therapy	50 (11.1)	183 (19.1)	< 0.001
RDS	30 (6.7)	146 (15.2)	< 0.001
BPD	10 (2.2)	78 (8.1)	< 0.001
TTN	13 (2.9)	21 (2.2)	0.424
IVH (≥ grade 3)	4 (0.9)	8 (0.8)	> 0.999
PVL	4 (0.9)	15 (1.6)	0.305
NEC (≥ stage 2)	2 (0.4)	18 (1.9)	0.034
ROP (≥ grade 3)	4 (0.9)	25 (2.6)	0.034
Hypoglycemia	16 (3.6)	35 (3.6)	0.933
Hyperbilirubinemia	60 (13.3)	164 (17.1)	0.073
Neonatal sepsis	9 (2.0)	28 (2.9)	0.315
Neonatal death	4 (0.9)	6 (0.6)	0.735

Data are presented as number (percentage) or mean ± standard deviation.

*Hb* hemoglobin, *NICU* neonatal intensive care unit, *RDS* respiratory distress syndrome, *BPD* bronchopulmonary dysplasia, *TTN* transient tachypnea of the newborn, *IVH* intraventricular hemorrhage, *PVL* periventricular leukomalacia, *NEC* necrotizing enterocolitis, *ROP* retinopathy of prematurity.

^a^Analyzed only in babies who had hemoglobin exam after birth (151 in the delayed cord clamping group and 433 in the immediate cord clamping group).

**Table 5:** Subgroup analyses of primary outcomes.

Outcome variables	Preterm			Term		
	DCC	ICC	*P-*value	DCC	ICC	*P-*value
Maternal	(n = 92)	(n = 278)		(n = 133)	(n = 202)	
Predelivery Hb level (g/dl)	11.6 ± 1.4	11.5 ± 1.5	0.649	11.9 ± 1.4	12.0 ± 1.4	0.838
Postpartum day 1 Hb level (g/dl)	10.1 ± 1.5	10.3 ± 1.6	0.159	10.4 ± 1.6	10.6 ± 1.6	0.170
Postpartum day 3 Hb level (g/dl)	9.7 ± 1.4	10.0 ± 1.6	0.210	10.1 ± 1.6	10.2 ± 1.5	0.554
Postpartum Hb drop ≥ 20%	43 (46.7)	89 (32.0)	0.011	49 (36.8)	72 (35.6)	0.823
Blood transfusion	9 (9.8)	26 (9.43)	0.903	11 (8.3)	9 (4.5)	0.149
Neonatal	(n = 184)	(n = 556)		(n = 266)	(n = 404)	
Hb level at birth (g/dl)^a^	16.9 ± 3.5	16.4 ± 2.6	0.139	19.5 ± 2.9	18.9 ± 2.8	0.450
Blood transfusion	13 (7.1)	78 (14.0)	0.013	2 (0.8)	6 (1.5)	0.488

Outcome variables	Vaginal delivery			Cesarean section		
	DCC	ICC	*P-*value	DCC	ICC	*P-*value
Maternal	(n = 37)	(n = 36)		(n = 188)	(n = 444)	
Predelivery Hb level (g/dl)	12.0 ± 1.3	12.0 ± 1.2	0.970	11.8 ± 1.4	11.7 ± 1.5	0.565
Postpartum day 1 Hb level (g/dl)	9.7 ± 1.7	9.5 ± 1.8	0.783	10.4 ± 1.5	10.5 ± 1.5	0.231
Postpartum day 3 Hb level (g/dl)	–	**–**	–	9.9 ± 1.5	10.1 ± 1.5	0.350
Postpartum Hb drop ≥ 20%	19 (51.4)	18 (50.0)	0.908	73 (38.8)	143 (32.2)	0.109
Blood transfusion	6 (16.2)	6 (16.7)	0.959	14 (7.4)	29 (6.5)	0.676
Neonatal	(n = 74)	(n = 72)		(n = 376)	(n = 888)	
Hb level at birth (g/dl)^a^	16.7 ± 2.4	16.8 ± 2.1	0.918	17.5 ± 3.7	16.6 ± 2.7	0.008
Blood transfusion	1 (1.4)	4 (5.6)	0.206	14 (3.7)	80 (9.0)	0.001

Outcome variables	Monochorionic			Dichorionic		
	DCC	ICC	*P-*value	DCC	ICC	*P-*value
Maternal	(n = 31)	(n = 76)		(n = 194)	(n = 404)	
Predelivery Hb level (g/dl)	12.1 ± 1.3	11.6 ± 1.6	0.103	11.8 ± 1.4	11.8 ± 1.5	0.920
Postpartum day 1 Hb level (g/dl)	10.4 ± 1.7	10.5 ± 1.6	0.870	10.2 ± 1.6	10.5 ± 1.6	0.100
Postpartum day 3 Hb level (g/dl)	9.9 ± 1.5	10.1 ± 1.5	0.596	9.9 ± 1.6	10.1 ± 1.6	0.423
Postpartum Hb drop ≥ 20%	13 (41.9)	21 (27.6)	0.149	79 (40.7)	140 (34.7)	0.149
Blood transfusion	0 (0)	8 (10.5)	0.102	20 (10.3)	27 (6.7)	0.123
Neonatal	(n = 62)	(n = 152)		(n = 388)	(n = 808)	
Hb level at birth (g/dl)^a^	18.2 ± 3.7	16.8 ± 3.4	0.054	17.2 ± 3.5	16.6 ± 2.6	0.072
Blood transfusion	6 (9.7)	12 (7.9)	0.670	9 (2.3)	72 (8.9)	< 0.001

*DCC* delayed cord clamping, *ICC* immediate cord clamping; Hb, hemoglobin.

^a^Analyzed only in babies who had hemoglobin exam after birth (preterm DCC 122, ICC 402; term DCC 29, ICC 31; vaginal delivery DCC 20, ICC group 31; cesarean section DCC 131, ICC 402; monochorionic DCC 32, ICC 64; dichorionic DCC 119, ICC 369).

**Table 6:** Subgroup analyses (1st twin and 2nd twin).

Outcome variables	Preterm			Term		
	DCC	ICC	*P-*value	DCC	ICC	*P-*value
1st twin	(n = 92)	(n = 278)		(n = 133)	(n = 202)	
Hb level at birth (g/dl)^a^	17.0 ± 3.3	16.3 ± 2.6	0.138	18.7 ± 3.0	19.2 ± 2.2	0.637
Blood transfusion	7 (7.6)	35 (12.6)	0.192	2 (1.5)	2 (1.0)	0.651
2nd twin	(n = 92)	(n = 278)		(n = 133)	(n = 202)	
Hb level at birth (g/dl)^b^	16.9 ± 3.7	16.6 ± 2.6	0.526	20.4 ± 2.6	18.6 ± 3.3	0.135
Blood transfusion	6 (6.5)	43 (15.5)	0.028	0 (0.0)	4 (2.0)	0.155

Outcome variables	Vaginal delivery			Cesarean section		
	DCC	ICC	*P-*value	DCC	ICC	*P-*value
1st twin	(n = 37)	(n = 36)		(n = 188)	(n = 444)	
Hb level at birth (g/dl)^a^	16.8 ± 2.6	16.4 ± 2.0	0.599	17.4 ± 3.5	16.5 ± 2.8	0.050
Blood transfusion	0 (0.0)	2 (5.6)	0.240	9 (4.8)	35 (7.9)	0.162
2nd twin	(n = 37)	(n = 36)		(n = 188)	(n = 444)	
Hb level at birth (g/dl) ^b^	16.6 ± 2.4	17.3 ± 2.2	0.467	17.7 ± 3.9	16.7 ± 2.7	0.069
Blood transfusion	1 (2.7)	2 (5.6)	0.615	5 (2.7)	45 (10.1)	0.001

Outcome variables	Monochorionic			Dichorionic		
	DCC	ICC	*P-*value	DCC	ICC	*P-*value
1st twin	(n = 31)	(n = 76)		(n = 194)	(n = 404)	
Hb level at birth (g/dl)^a^	18.9 ± 3.5	16.9 ± 3.4	0.068	16.9 ± 3.2	16.4 ± 2.6	0.251
Blood transfusion	3 (9.7)	4 (5.3)	0.411	6 (3.1)	33 (8.2)	0.019
2nd twin	(n = 31)	(n = 76)		(n = 194)	(n = 404)	
Hb level at birth (g/dl)^b^	17.6 ± 3.9	16.7 ± 3.5	0.388	17.5 ± 3.7	16.7 ± 2.5	0.156
Blood transfusion	3 (9.7)	8 (10.5)	> 0.999	3 (1.5)	39 (9.7)	< 0.001

*DCC* delayed cord clamping, *ICC* immediate cord clamping; Hb, hemoglobin.

^a^Analyzed only in babies who had hemoglobin exam after birth (preterm DCC 63, ICC 200; term DCC 16, ICC 15; vaginal delivery DCC 11, ICC group 17; cesarean section DCC 68, ICC 198; monochorionic DCC 16, ICC 30; dichorionic DCC 63, ICC 185).

^b^Analyzed only in babies who had hemoglobin exam after birth (preterm DCC 59, ICC 202; term DCC 13, ICC 16; vaginal delivery DCC 9, ICC group 14; cesarean section DCC 63, ICC 204; monochorionic DCC 16, ICC 34; dichorionic DCC 56, ICC 184).

There was no significant difference in the rate of IVH (≥ grade 3), PVL, hypoglycemia, hyperbilirubinemia, sepsis, or death between the two groups (Table 4). The rate of RDS was significantly lower in the DCC group than in the ICC group (DCC vs. ICC: 6.7% vs. 15.2%, P < 0.001, adjusted odds ratio: 0.517, 95% confidence interval: 0.276, 0.970) after controlling for parity, PPROM, chorionicity, antenatal corticosteroids treatment, gestational age at delivery, cesarean section, twin birth order, sex, birth weight, and SGA (Table 7). Rates of NICU admission, mechanical ventilator therapy, BPD, NEC (≥ stage 2), and ROP (≥ stage 3) were lower in the DCC group than in the ICC group. However, these were not statistically significant in the multivariable analysis (Table 7).

**Table 7:** Multivariable regression analysis of delayed cord clamping vs. immediate cord clamping.

Outcome variables^a^	aOR	95% CI	*P-*value
NICU admission	1.243	0.854, 1.810	0.257
Mechanical ventilator therapy	0.946	0.578, 1.550	0.828
RDS	0.523	0.279, 0.981	0.043
BPD	0.485	0.173, 1.360	0.169
NEC (≥ stage 2)	0.435	0.068, 2.764	0.377
ROP (≥ grade 3)	2.758	0.571, 13.322	0.207

*aOR* adjusted odds ratio, *CI* confidence interval, *NICU* neonatal intensive care unit, *RDS* respiratory distress syndrome, *BPD* bronchopulmonary dysplasia, *NEC* necrotizing enterocolitis, *ROP* retinopathy of prematurity.

^a^Adjusted for multiparity, preterm premature rupture of membranes, antenatal corticosteroids treatment, chorionicity, gestational age at delivery, cesarean section, twin birth order, sex, birth weight, and small-for-gestational age.

## Discussion

In this study, we evaluated maternal and neonatal outcomes following DCC versus immediate cord clamping (ICC) in twin pregnancies. Our study demonstrated that DCC in twin pregnancy did not result in increased maternal postpartum blood loss, although it was associated with significantly higher neonatal hemoglobin levels, lower neonatal blood transfusion rate, and reduced risk of RDS compared with ICC.

Although DCC is known to be more beneficial for neonates than traditional ICC, DCC might also have potential risks of maternal or neonatal adverse effects such as increased maternal blood loss, neonatal polycythemia, hyperbilirubinemia, and jaundice^1,11,17,20^. As for maternal bleeding complications, theoretically, DCC might potentially cause more maternal blood loss at the uterine incision site or episiotomy site. In particular, DCC might result in greater maternal blood loss in twin pregnancies because DCC takes even longer time from delivery to incision site repair than single pregnancies.

In previous studies of singleton pregnancies, DCC did not increase the risk of maternal bleeding complications such as increased estimated blood loss, higher postpartum hemorrhage rate, higher maternal blood transfusion rate, lower postpartum hemoglobin levels, or greater mean hemoglobin changes^1,2^. In a randomized controlled trial of multiple-birth infants born preterm at 28‒36 weeks of gestation, rate of postpartum hemorrhage (defined as an estimated blood loss of > 500 ml for vaginal delivery or > 1000 mL for cesarean delivery) was significantly higher in the DCC group than the ICC group (6/24 [25%] in DCC vs. 1/23 [4.3%] in ICC; P = 0.04)^7^. However, the sample size of that study was too small (24 and 23 mothers in DCC and ICC groups, respectively), with triplet pregnancies (7/47) included in that study. In a retrospective cohort study including 449 multiple pregnancies, there were no significant differences in maternal bleeding complications (including postpartum hemorrhage, estimated blood loss, maternal blood transfusions, therapeutic hysterectomy) between DCC and ICC groups^13^. That study was similar to our study because the sample size was large, the rate of higher-order pregnancies was relatively low (2/154 in DCC vs. 9/295 in ICC), and both preterm and full-term infants were included. In our study, postpartum hemoglobin levels tended to be slightly lower in the DCC group than in the ICC group, although such differences were not statistically different. In addition, the rate of postpartum hemoglobin drop ≥ 20% was slightly higher in the DCC group than in the ICC group. It was only statistically significant in the preterm delivery group. However, severe maternal postpartum hemorrhage leading to blood transfusion was not significantly different among all subgroups except the preterm delivery group. The reason for a higher rate of postpartum hemoglobin drop ≥ 20% in the preterm DCC group is not certain. It might be explained that most of the twin deliveries at preterm gestation are performed emergently and lower uterine segment is underdeveloped at preterm gestation which can result in more bleeding at the uterine incision site during cesarean section, especially when the uterine closure is delayed due to DCC. Another retrospective study of 82 twin pregnancies delivered at < 32 weeks of gestation showed that DCC was associated with higher estimated maternal blood loss in the cesarean section group, although maternal complications including maternal hemoglobin decrease, postpartum hemorrhage, blood transfusions, and hysterectomy were comparable between DCC and ICC groups^6^.

Previous studies have well demonstrated that DCC could increase neonatal hemoglobin levels, improve iron status, and reduce risk of neonatal morbidities in singleton pregnancies^1,3,4,8^. However, the effect of DCC on neonatal hemoglobin level in twin pregnancies was controversial in previous studies. A randomized controlled trial showed that neonatal hemoglobin levels were similar in preterm twins or triplets infants who received DCC (n = 51) or ICC (n = 50)^7^. A retrospective study of dichorionic twin pregnancies at 23‒32 weeks of gestation also reported that neonates who received DCC had no difference in neonatal hemoglobin level^14^. However, only 8 twin pregnancies (16 neonates) were included in the DCC group. In other studies, twins who received DCC had higher hemoglobin levels but lower rates of blood transfusion^6,12,15,16^. In our study, mean neonatal hemoglobin level was higher while neonatal blood transfusion rate was significantly lower in the DCC group than in the ICC group in all study population. In subgroup analyses, neonatal hemoglobin levels in all subgroups tended to be higher in the DCC group than in the ICC group. However, neonatal hemoglobin level was significant only in the cesarean section group. This was probably because the sample size of our study, especially the numbers of subjects in each subgroup, was too small. In our institute, we do not perform routine complete blood cell count test for healthy babies who are not admitted to the NICU. Thus, neonatal hemoglobin level data were available in only 151 (33.6%) of 450 in the DCC group and 433 (45.2%) of 962 in the ICC group. This is one of the main limitations of our study. However, neonatal blood transfusion data were available in all neonates. Its rate was significantly lower in the DCC group than in the ICC group, especially in twins delivered at preterm, twins delivered by cesarean section, and dichorionic twins.

Other benefits of DCC include reduced risk neonatal morbidities such as respiratory distress syndrome, intraventricular hemorrhage, necrotizing enterocolitis, and death or major disability^1,3–5,16,21^. However, most of these studies were done in singleton pregnancies. There are only a few studies on twin pregnancies^6,7,12,14–16^, and the results were controversial. A prospective cohort study of 202 twin pregnancies at > 32 weeks of gestation showed that twins who received DCC were at a lower risk of respiratory disorders and NICU admission^16^. Another retrospective cohort study including twin pregnancies at < 30 weeks of gestation showed a shorter NICU length of stay in DCC twins^15^. However, other studies of twin pregnancies found no differences in mechanical ventilator treatment, RDS, BPD, IVH, NEC, ROP, sepsis, death, or severe neurologic injury^6,7,12,14^. In our study, rates of NICU admission, mechanical ventilator treatment, RDS, BPD, NEC, and ROP were lower in the DCC group than in the ICC group, but this may be due to a lower rate of PTD in the DCC group. The rates of NICU admission, mechanical ventilator treatment, BPD, NEC, and ROP were not significantly different between the two groups after adjusting for confounding variables including gestational age at delivery. However, the rate of RDS was significantly lower in the DCC group in the multivariable analysis. The exact reason for reduced risk of RDS in babies who received DCC is unclear. Previous studies have suggested that DCC may improve hemodynamic stability and reduce complications related to poor oxygenation^22–26^. Chiruvolu et al. reported that the incidence of RDS and surfactant administration decreased when DCC is performed in preterm singleton pregnancies^4,5^. They explained that spontaneous respiration resulting in lung aeration starts during DCC, and thus the need for resuscitation decreases due to better cardiopulmonary transition to extrauterine life. Especially in premature infants, gas exchange is difficult due to high pulmonary vascular resistance^27,28^. In a study using the computational lumped parameter model of the placental and respiratory system, it was found that preterm infants who received ICC had lower blood volume, lower cardiac output, and lower blood pressure, whereas preterm infants who received DCC had higher oxygen saturation of the carotid and pulmonary arteries immediately after birth when DCC was performed^29^. However, the sample size of our study as well as other previous studies might be insufficient to conclude effects on neonatal outcomes other than neonatal hematologic effects in twin pregnancies.

The strength of this study was that it had a relatively large sample size of 705 twin pregnant women (1410 neonates) compared to previous twin studies. Another strength was that we recruited all twin pregnancies including both preterm and term gestation, both monochorionic and dichorionic twins, and both vaginal and cesarean delivery, while previous studies only included preterm twins or dichorionic twins. However, our sample size was still insufficient to have an adequate power because numbers of subjects in each subgroup were too low. In addition, neonatal hemoglobin level data were available in only less than half of all twin neonates. This study is further limited by the inherent nature of a retrospective study design. Maternal baseline characteristics and pregnancy outcomes were not comparable. Especially, more preterm twin pregnancies were included in the ICC group and cesarean section rate was higher in the ICC group. Although we performed subgroup analyses and multivariable analyses to control for bias, there might be other unknown potential confounding factors including individual surgeons’ operative skill. There was no common protocol of umbilical cord clamping in our institute and DCC or ICC was performed at physicians’ discretion. Individual surgeons’ operative skills might be one of the unknown potential confounding factors. However, we were not able to adjust this because there were more than 30 doctors (including residents, fellows and professors) who delivered babies (vaginal or cesarean) during 8 years of study period.

## Conclusion

Delayed umbilical cord clamping in twin pregnancy was not associated with maternal postpartum bleeding complications, although it was associated with increased neonatal hemoglobin level and decreased risk of RDS. However, more well-designed studies with larger sample size are needed to identify the benefit and risk of DCC in twin pregnancies.

## Data Availability

All datasets used and/or analyzed during the current study are available from the corresponding author upon reasonable request.
